# Editorial: Advancing human wellbeing: environment-focused AI technologies

**DOI:** 10.3389/frai.2026.1882773

**Published:** 2026-06-24

**Authors:** Najlae Idrissi, Mathieu Roche, Gurjot Singh Gaba, Yassine Sadqi, Mohammed Erritali

**Affiliations:** 1 Intelligence, Data and Computing Team (IDCT), Faculty of Sciences and Techniques, Sultan Moulay Slimane University, Beni Mellal, Morocco; 2 CIRAD, UMR TETIS, Montpellier, France; 3 TETIS, Univ. de Montpellier, AgroParisTech, CIRAD, INRAE, Montpellier, France; 4 Department of Computer and Information Science (IDA), Linköping University (LiU), Linköping, Sweden; 5 Laboratory L2IS, Faculty of Sciences and Techniques, Cadi Ayyad University (UCA), Marrakesh, Morocco

**Keywords:** computer vision, deep learning, human-computer interaction, large language models, text mining, artificial intelligence, healthcare, sentiment analysis

## 1 Introduction

Artificial intelligence is increasingly influencing modern research and is becoming ever more positioned at the intersection of scientific and technological innovation, environmental sustainability, and human development. By offering new methods for processing, analyzing, and interpreting complex data, it has enabled its rapid adoption in fields such as healthcare (Faiyazuddin et al., 2025), computerized sentiment detection (Yaqoob et al., 2026; Lima et al., 2025), pharmacology and drug safety (Gérard et al., 2026; Domingo, 2026), and any area involving data-driven decision-making (Salehi and Babaei, 2026). AI is currently regarded as a powerful catalyst for solutions that address complex environmental challenges whilst enhancing human wellbeing. This dual role is helping to shift the focus of AI solutions toward human-centered AI systems, whose aim is not only to ensure efficiency and automation, but to produce eco-responsible systems that improve quality of life in various social contexts.

The relation between AI, environmental systems, and human wellbeing remains complex and multifaceted. Although AI approaches offer considerable opportunities to optimize resource management and enable smart environmental monitoring, it also poses concerns regarding energy consumption, environmental footprint, and unequal socio-economic impacts. Recent research shows that the effects of AI on environmental sustainability and wellbeing are not equal. For example, in areas such as health and productivity, the benefits are often counterbalanced by potential risks related to inequality, social cohesion, and environmental impact. This opens the way for interdisciplinary studies and research on how AI systems can be designed and deployed in a responsible manner to ensure balanced results and equity in their application.

Finally, this Research Topic includes three original articles, each highlighting the convergence between artificial intelligence and human wellbeing. From deep learning approaches for contextual emotional detection and medical image analysis to AI-assisted literature mining for hepatotoxicity assessment, these studies show intelligent systems can support healthcare, enhance decision-making, and improve quality of life through data-driven and human-centered applications.

## 2 Human wellbeing and AI technologies

The Research Topic is organized into three main themes as follows: AI for Emotion Detection, AI for Toxicological Research, and AI for Medical Imaging. Each theme is represented by a single contribution.

### 2.1 Deep learning for emotion detection

In the context of exploring contextual emotion recognition, Limami et al. proposed a deep learning system by combining DCNN and VGG19 architectures, enhanced through the fusion of discrete emotional categories and continuous emotional dimensions. Their approach has shown significant improvements compared to prior methods in state-of-the-art, highlighting the importance of contextual information for emotional computing.

### 2.2 AI language models for toxicological research

The volume of toxicology research is increasing rapidly, posing considerable challenges to researchers in analyzing and synthesizing the growing body of literature. This underscores the need for more effective knowledge management approaches. In this context, Bauer et al. propose a hybrid framework combining text mining, word embeddings, and large language models to calculate a hepatotoxicity score. The findings highlight the relevance of AI-driven literature mining, in particular through LLMs, to support drug safety assessment and toxicological research.

### 2.3 Deep learning for medical imaging

Anand and Khajuria investigated the effectiveness of transformer-based (Swin Transformer V2) and modern convolutional neural network (ConvNeXt V2) for breast cancer classification using histopathological images. Results demonstrate that transformer-based models significantly outperform the CNN-based approach, reinforcing the potential of visual transformers to advance diagnosis in medical imaging.

## 3 Conclusion

The interaction between AI technologies and the environment, with a focus on the human, has become a fundamental area of research. The published articles on this topic contribute to advancing human-centered research, as reflected by the strong interest of the community with more than 13,000 views and downloads.
